# System analysis identifies UBE2C as a novel oncogene target for adrenocortical carcinoma

**DOI:** 10.1371/journal.pone.0289418

**Published:** 2023-08-03

**Authors:** Renlun Huang, Lang Guo, Chiwei Chen, Yuyang Xiang, Guohao Li, Jieyan Zheng, Yanping Wu, Xiu Yuan, Jianfu Zhou, Wenxi Gao, Songtao Xiang

**Affiliations:** 1 The Research Center of Integrative Cancer Medicine, Discipline of Integrated Chinese and Western Medicine, The Second Clinical College of Guangzhou University of Chinese Medicine, Guangzhou, Guangdong, China; 2 Department of Urology, Guangdong Provincial Hospital of Chinese Medicine, Guangzhou, Guangdong, China; 3 Department of Urology, Hubei Provincial Hospital of Traditional Chinese Medicine, Hubei University of Chinese Medicine Affiliated Hubei Hospital of Chinese Medicine, Wuhan, Hubei, China; 4 The Second Clinical College of Southern Medical University, Guangzhou, Guangdong, China; 5 First Clinical College and Affiliated Hospital, Hubei University of Traditional Chinese Medicine, Wuhan, Hubei, China; 6 Liyuan Hospital, Tongji Medical College, Huazhong University of Science and Technology, Wuhan, Hubei, China; The University of Alabama at Birmingham (UAB), UNITED STATES

## Abstract

Ubiquitin Conjugating Enzyme 2C (UBE2C) is an emerging target gene for tumor progression. However, the tumorigenic effect and mechanism of UBE2C in adrenocortical carcinoma (ACC) remains unclear. Systematic investigation of the tumorigenic effect of UBE2C may help in understanding its prognostic value in adrenocortical carcinoma. First, we exploited the intersection on DFS-related genes, OS-related genes, highly expressed genes in adrenocortical carcinoma as well as differentially expressed genes (DEGs) between tumor and normal, and then obtained 20 candidate genes. UBE2C was identified to be the most significant DEG between tumor and normal. It is confirmed that high expression of UBE2C was strongly associated with poor prognosis in patients with ACC by analyzing RNA-seq data of ACC obtained from the Cancer Genome Atlas (TCGA) database implemented by *ACLBI Web-based Tools*. UBE2C expression could also promote m6A modification and stemness in ACC. We found that UBE2C expression is positively associated with the expression of CDC20, CDK1, and CCNA2 using *ACLBI Web-based Tools*, indicated the hyperactive cell cycle progression present in ACC with high UBE2C expression. In addition, UBE2C knockdown could significantly inhibit the proliferation, migration, invasion, EMT of adrenocortical carcinoma cells as well as the cell cycle progression in vitro. Notably, pan-cancer analysis also identified UBE2C as an oncogene in various tumors. Taken together, UBE2C was strongly associated with poor prognosis of patients with ACC by promoting cell cycle progression and EMT. This study provides a new theoretical basis for the development of UBE2C as a molecular target for the treatment of ACC.

## Introduction

Adrenocortical carcinoma (ACC) is an aggressive tumor originating in the adrenocortical cortex with a very low incidence about 0.5–1 per 1 million people [1] and poor prognosis with 5-year overall survival ranging from 16% to 47% [1, 2]. The 5-year survival rate of ACC at stage I is about 81%, but when the disease progresses to stage Ⅳ, the 5-year survival rate can be reduced to about 13% [3]. Disease progression is the main reason affecting the prognosis of ACC. Radical surgical excision is the only cure option for primary ACC [4]. However, the recurrence rate of ACC after surgical excision is highly [5]. Although it is prone to metabolic and endocrine toxicity, mitotane remain as the only drug approved for the treatment of advanced adrenocortical carcinoma and postoperative [6]. It follows that there is a lack of other effective drug for the treatment of ACC, so it is urgent to find new therapeutic targets. Hence, deciphering the complex molecular composition of ACC progression and developing targeted therapeutic agents, bioinformatics analysis as a convenient and effective strategy is a good choice to screen the available therapeutic targets.

Ubiquitination of modified proteins is an important cellular mechanism that targets the degradation of abnormal or short-lived proteins and plays an important role in tumor progression [7]. UBE2C is a key member of the E2 ubiquitin-binding enzyme family, encoding proteins necessary for the destruction of target proteins [8]. Accumulating evidence suggested that overexpression of UBE2C can promote the proliferation of ACC by promoting the G2/M phase transition [9] and the activation of the Spindle and Kinechore-Associated (SKA) Complex [10]. Notably, the KRAS^G12D^ mutation can promote the expression of UBE2C to promote cell cycle progression and autophagy in lung cancer, which indicated that UBE2C is an attractive carcinogenic genetic target for lung cancer with KRAS mutations [11]. A comprehensive bioinformatics analysis indicated the effect of UBE2C in promoting tumor progression [12], but the carcinogenic effect of UBE2C in ACC still needs to be further investigated.

In this study, we confirmed that UBE2C is a major response gene that affects the prognosis of ACC. In addition, we also explored the relationship between UBE2C expression and m6A methylation, proliferation-related and metastasis-related pathways in ACC. Moreover, in vitro experiments showed that UBE2C knockdown result in poor prognosis of ACC by inhibiting the proliferation, migration, invasion, epithelial–mesenchymal transition (EMT) of adrenocortical carcinoma cells as well as the cell cycle progression. And the pan-cancer analysis suggested that UBE2C is an oncogenic gene in various tumors. In conclusion, this study identified UBE2C as a novel oncogenic gene in promoting adrenocortical carcinoma progression and contributing to its poor prognosis.

## Materials and methods

### Research framework

**Fig 1:**
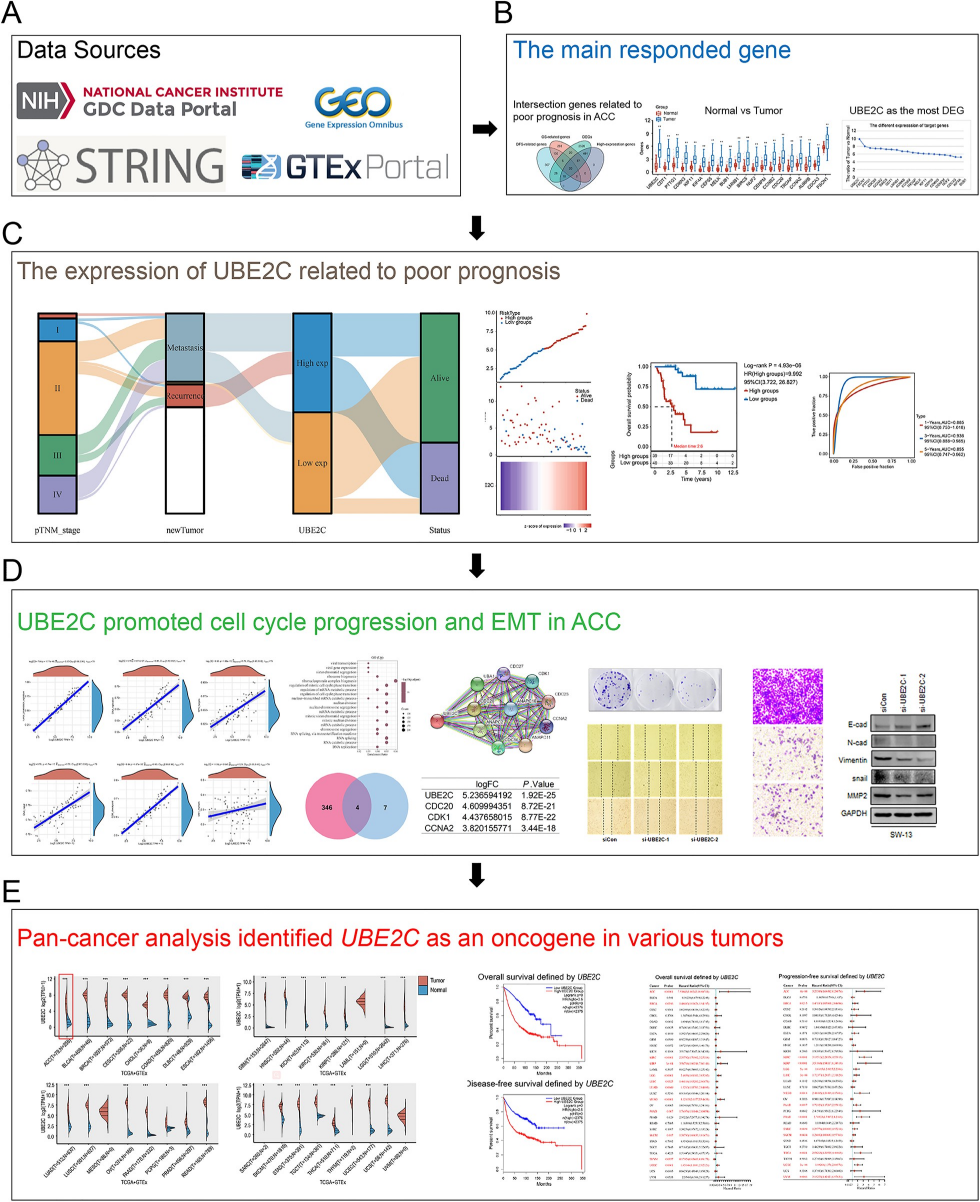
Overview of the research design. (A) Data sources used in this research; (B) The main response genes related to poor prognosis in ACC were analyzed. (C) The expression of UBE2C is positively correlated with advanced clinicopathological characteristics and poor prognosis of ACC. (D) UBE2C expression can promote cell cycle progression and EMT. (E) Pan-cancer analysis identified UBE2C as an oncogene in various tumors.

Fig 1 showed the framework of this study.

### Data retrieval, preprocessing, and analysis

Obtained from TCGA database (https://tcga-data.nci.nih.gov/tcga/), GEO database (http://www.ncbi.nlm.nih.gov/geo), GTEx database (https://www.gtexportal.org/), these significant data were used to detect target genes related to poor prognosis in patients with ACC. These data were analyzed using R packages implemented by R version 4.0.3 (R foundation for statistical computing, 2020). Conveniently, all above data retrieval, preprocessing, and analysis were performed in STRING (https://cn.string-db.org/), *ACLBI Web-based Tools* (https://www.aclbi.com/) or *Xiantao Academic web-based Tools* (https://www.xiantao.love/products).

### Venn diagrams

In order to screen out the likely response gene, Venn diagrams were created to analyze the difference between several dataset using a web-based tool (http://www.bioinformatics.com.cn/static/others/jvenn/example.html).

### mRNA expression-based stemness index (mRNAsi)

mRNA expression-based stemness index (mRNAsi) was identified as a measure of the tumor development according to the previously publication-n [13]. Based on the OCLR algorithm [13], the mRNAsi of ACC was calculated using ACC RNA-seq data obtained from TCGA database implemented by *ACLBI Web-based Tools*.

### Cell culture

The ACC cell line SW-13 was purchased from the American Type Culture Collection (ATCC, Maryland, USA) and cultured in Leibovitz’s L-15 Medium (L-15, 11415064, Gibco, Grand Island, NY, USA) supplemented with 1% penicillin and streptomycin and 10% fetal bovine serum (Gibco). The culture of SW-13 cells needed to maintain at 37°C.

### RNA interference

RNA interference (RNAi) was used to interfere with the expression of UBE2C in SW-13 cells. The siRNAs targeting UBE2C (TranSheepBio, Shanghai, China) and the scramble siRNAs were purchased. And then siRNAs targeting UBE2C were transfected into SW-13 cells using Lipofectamine™ 3000 (L3000015, ThermoFisher SCIENTIFIC) according to the manufacturer’s instructions.

### Cells counting

Cells proliferation was assessed using Neubauer counting chamber. First, RNAi technology was used to interfere with the UBE2C expression in SW-13 cells. Second, the treated SW-13 cells and non-treated SW-13 cells were seeded into the 6-well plates. Then, the SW-13 cells were digested with trypsin and resuspended in PBS. Finally, the proliferation of SW-13 cells was assessed by cell counting using Neubauer counting chamber.

### Colony formation assay

The effect of UBE2C on the colony formation capabilities of SW-13 cells was detected as previously described [14]. Briefly, the expression of UBE2C in SW-13 cells were interfered using RNAi technology. The non-treated SW-13 cells and treated SW-13 cells (500 cells/well) were planted in a 6-well plate. The resultant colonies were fixed with 4% paraformaldehyde, and then coomassie blue solution were used to fix and stain the resultant colonies.

### Wound-healing assay and transwell assay

Wound-healing assay and transwell assay were performed as previously described [14] to detect the change of migration and invasion capabilities of SW-13 cells, respectively. In wound-healing assay, SW-13 cells in a logarithmic growth phase were collected to seeded in a 6-well plate. A wound was created mechanically using a 1 ml pipette tip. In transwell assay, the basement membrane was simulated using matrigel (354248, Corning, NY, USA).

### Western blotting

Western blotting was carried out as previously reported [15]. The antibodies used for western blotting included C-myc (18583S, CST), Cyclin D1 (55506S, CST), UBE2C (14234S, CST), cleased-PARP-1 (5625S, CST), cleased-Caspase-7 (9491T, CST), E-cad (20874-1-AP, proteintech), N-cad (22018-1-AP, proteintech), Vimentin (10366-1-AP, proteintech), snail (ab216347, abcam), MMP2 (ab92536, abcam), GAPDH (ab8245, abcam).

### Statistical analyses

Statistical analyses in this study were carried out using the SPSS 24.0 software (Abbott Laboratories, Chicago, USA). Data were shown as mean ± SD. *P*-values less than 0.05 was identified to be statistically significant.

## Results

### UBE2C expression strongly associated with advanced histopathological characteristics and poor prognosis of adrenocortical carcinoma (ACC)

**Fig 2:**
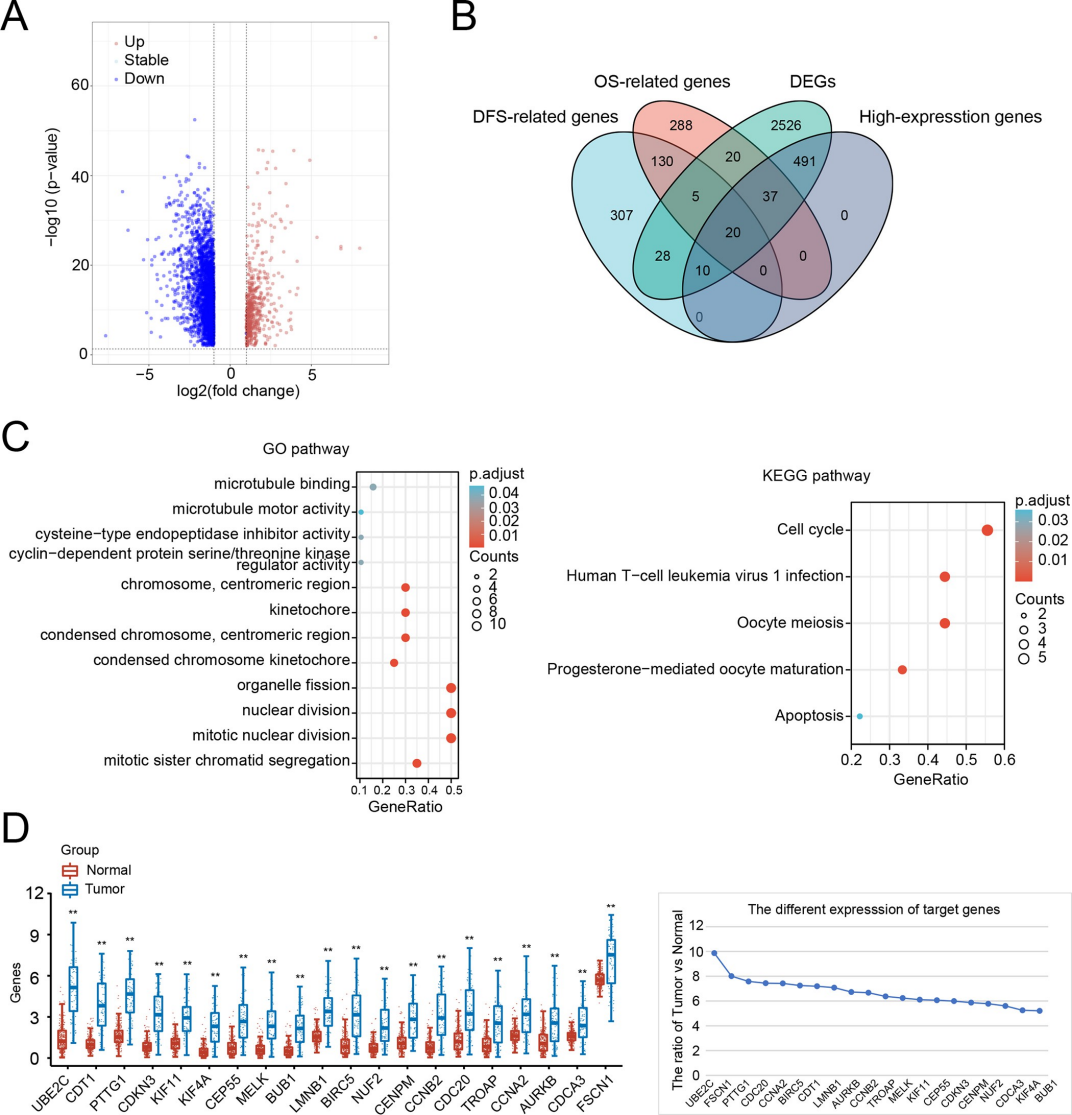
Differentially expressed genes in ACC and functional enrichment analysis. (A) Volcano plot: blue dots point different down-regulated genes and red dots point different up-regulated genes in ACC; (B) Venn diagrams: intersected with the DEGs, High-expression genes, OS-related genes, and DFS-related genes in ACC; (C) GO enrichment analysis and KEGG enrichment analysis of the 20 candidate genes; (D) the expression comparison of the 20 candidate genes between normal obtained for GTEx database and tumor obtained for TCGA database. *P < 0.05, **P < 0.01, ***P < 0.001, the asterisk represents the degree of statistical significance (*P).

**Fig 3:**
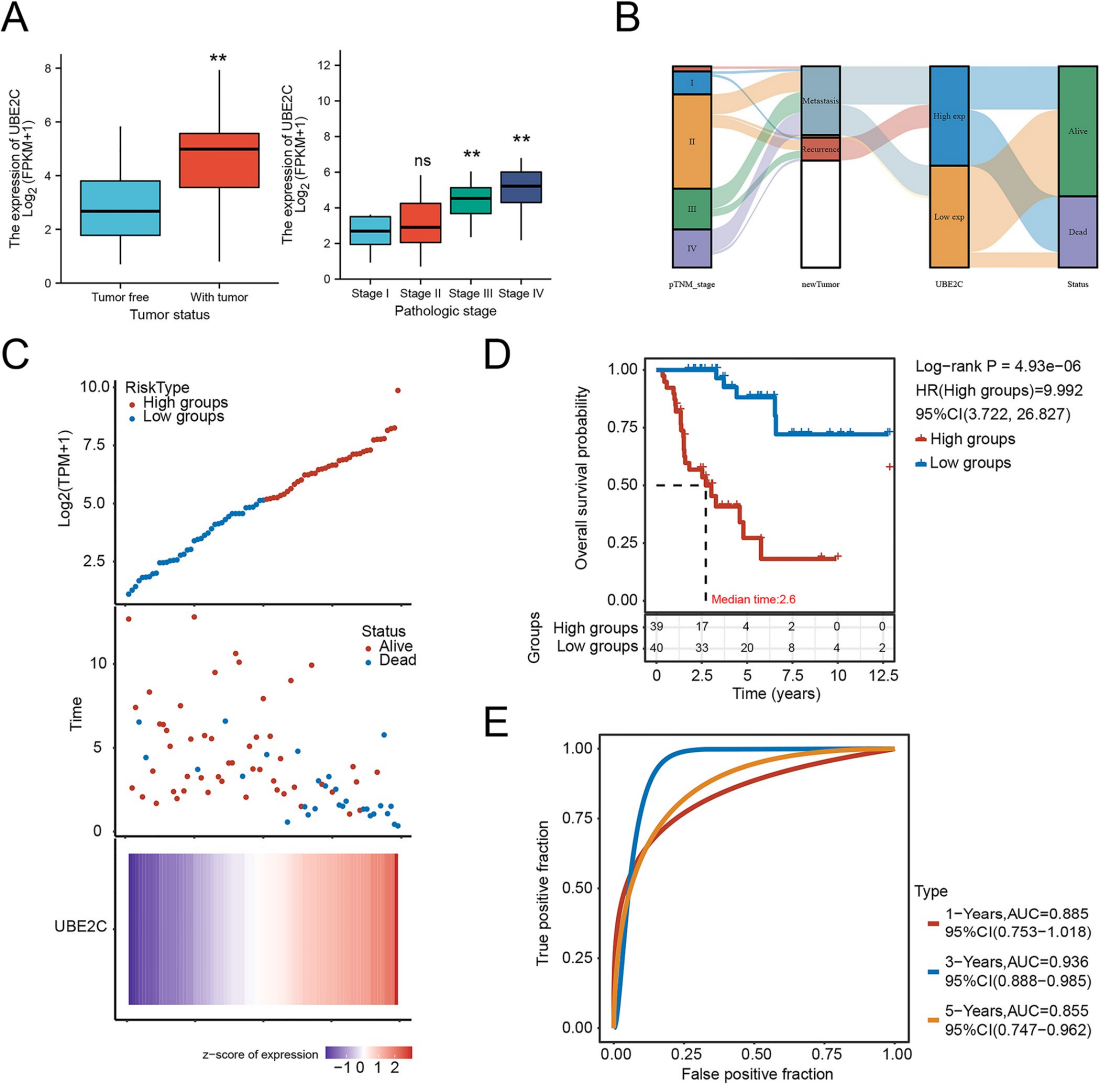
UBE2C expression indicated advanced clinical characteristics in ACC. (A) the expression of UBE2C in different tumor status and different pathologic stages; (B) Sankey diagram: each column represents a characteristic variable, different colors represent different types or stages, and the lines represent the distribution of the same sample in different characteristic variables; (C) the relationship between UBE2C expression and survival time as well as survival status in ACC; (D) the KM survival curve of the UBE2C in ACC; (E) the ROC curve and AUC value of the UBE2C at different times in ACC. *P < 0.05, **P < 0.01, ***P < 0.001, the asterisk represents the degree of statistical significance (*P).

In order to detect the main response gene strongly associated with poor prognosis in patients with ACC, we analyzed the intersection of four gene clusters including DFS-relates genes, OS-relates genes, differentially expressed genes (DEGs), and highly expressed genes. The volcano plot showed the DEGs of the patients with ACC (Fig 2A and S1 Table). After exploiting the intersection, we obtained 20 candidate genes in ACC including *CDC20*, *NUF2*, *BUB1*, *CCNA2*, *LMNB1*, *PTTG1*, *FSCN1*, *MELK*, *KIF11*, *CEP55*, *CDCA3*, *TROAP*, *CDKN3*, *CCNB2*, *CDT1*, *AURKB*, *CDT1*, *AURKB*, *BIRC5*, *UBE2C*, *CENPM*, *KIF4A* (Fig 2B and S2 Table). To identify the major response gene affecting prognosis in patients with ACC, GO and KEGG pathways enrichment analysis were used to analyze the activated signaling pathways accompanied by the highly expression of these candidate genes (Fig 2C). Notably, UBE2C was the most significant DEG between ACC and normal (Fig 2D and S3 Table). We also found that UBE2C was highly expressed in ACC especially in stage Ⅲ and stage Ⅳ using *Xiantao Academic Web-based Tools* (Fig 3A). To further study the correlation between UBE2C expression and histopathological characteristics of ACC, we tested UBE2C within the ACC dataset to better describe its functionality (Fig 3B and S4 Table). These analyses confirmed that ACC patients with high UBE2C expression tended to have a poor prognosis compared to those with low UBE2C expression (Fig 3C–3E). Taken together, UBE2C was highly expressed and strongly correlated with poor prognosis in patients with ACC.

### UBE2C expression promoted m^6^A methylation and stemness in ACC

**Fig 4:**
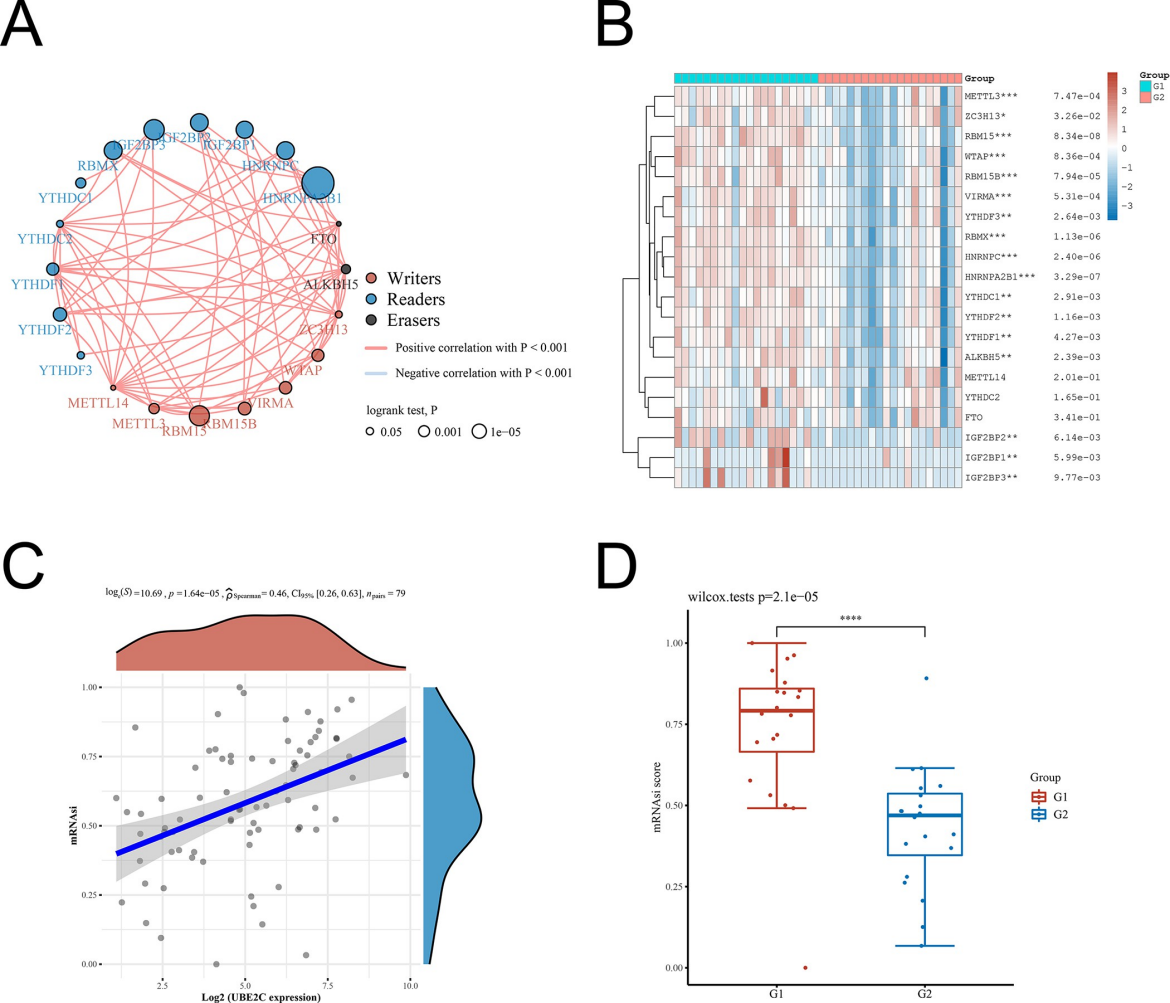
UBE2C expression promoted m6A methylation and stemness in ACC. (A) The correlation of expression on 20 m6A regulators in ACC. The size of each circle represents the prognosis of each m6A regulator and scaled through P-value. The m6A regulating protein of three RNA modifies is represented by different colors of circles. The line connecting the m6A regulators represents the interaction between them. (B) Heatmap: A heatmap of m6A-related gene expression, in which different color represent expression trends in different groups. G1 group was defined as above 75th percentile of UNE2C expression, and G2 group was defined as below 25th percentile of UNE2C expression. (C) The correlation between UBE2C expression and mRNAsi of ACC. (D) As described above, comparison of mRNAsi between G1 group and G2 group. *P < 0.05, **P < 0.01, ***P < 0.001, the asterisk represents the degree of statistical significance (*P).

In order to understand the mode of UBE2C regulating downstream pathway, we explored the correlation between UBE2C expression and m6A methylation in ACC. Based on the identification and analysis of the m6A methylation regulators by Juan Xu’s research [16], a circle was used to describe the interaction of expression on 20 m6A regulators in ACC using *ACLBI Web-based Tools* (Fig 4A). And then the expression levels of UBE2C were categorized into two groups including G1 group (above 75th percentile of UBE2C expression) and G2 group (below 25th percentile of UBE2C expression). The result showed that G1 group contained 16 significantly overexpressed m6A regulators compared with G2 group (Fig 4B and S5 Table). We found that UBE2C expression was positively associated with the mRNAsi in ACC using *ACLBI Web-based Tools*, which suggested that UBE2C may lead to poor prognosis in patients with ACC via promoting self-renewal of ACC stem cells (Fig 4C and 4D).

### UBE2C expression activated the molecular pathways of cell cycle, proliferation, and metastasis

**Fig 5:**
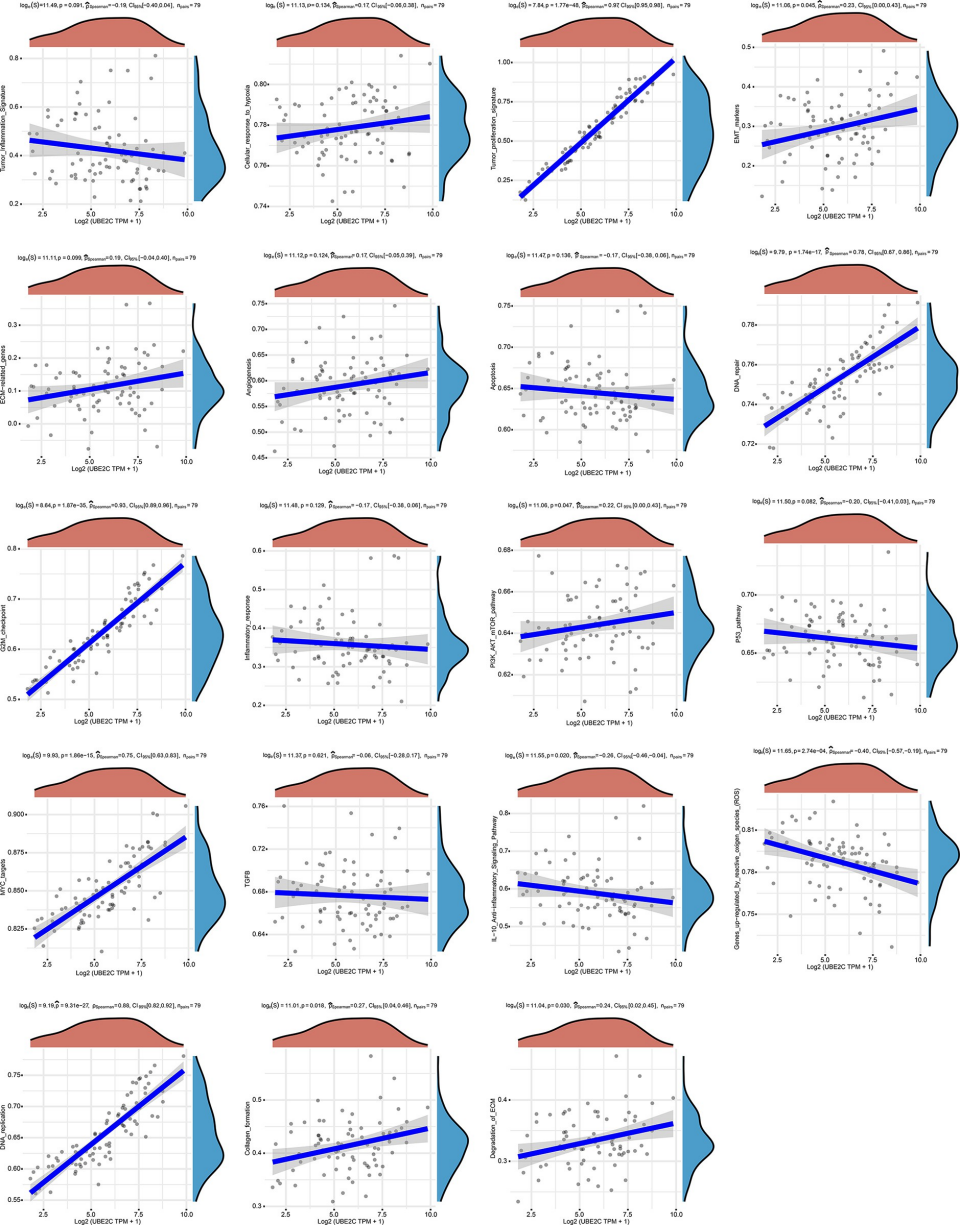
UBE2C expression was correlated with the activation of several signal pathway in ACC.

**Fig 6:**
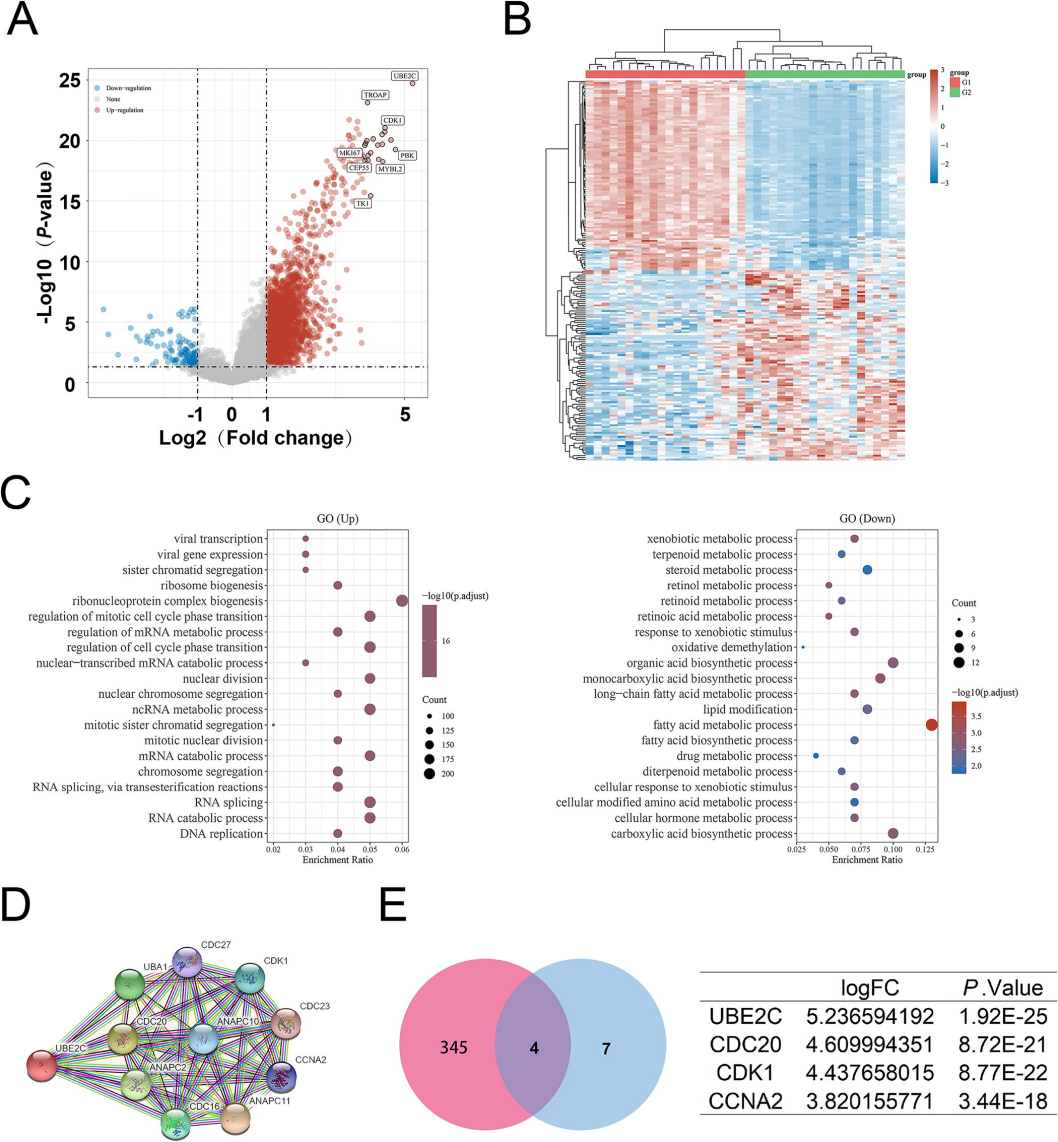
UBE2C expression with the activation of the molecular pathways of cell cycle, proliferation, and metastasis. (A) Volcano plot: blue dots point different down-regulated genes and red dots point different up-regulated genes; (B) Heatmap: DEGs between G1 group and G2 group. G1 group was defined as above 75th percentile of UNE2C expression, and G2 group was defined as below 25th percentile of UNE2C expression as described above; (C) GO enrichment analysis of these genes; (D) 10 candidate proteins related to UBE2C protein were identified using STRING database; (E) Venn diagrams: the intersection between 11 candidate proteins obtained from STRING database and 349 candidate genes obtained from significant DEGs between G1 group and G2 group.

At first, a previously published geneset [17] was analyzed for understanding the correlation between UBE2C expression and the activation of several signaling pathway in ACC implemented by *ACLBI Web-based Tools*, and the results showed that UBE2C expression was positively correlated with the activation of cell cycle, proliferation, and metastasis (Fig 5). To further explore the downstream signaling pathways activated by UBE2C, we analyzed DEGs between G1 group and G2 group as described above (Fig 6A–6C and S6 Table). Based on STRING protein-protein interaction network [18], we found that there are 10 candidate proteins closely related to UBE2C protein expression (Fig 6D). And based on LogFC and *P*-value (LogFC ≥ 2 or LogFC ≤ -2, P-value < 0.05), we identified 349 significantly DEGs (Fig 6E). And then, we screened out a cluster containing 4 genes related to cell cycle by intersecting the two data sets (Fig 6E and S7 Table).

### Pan-cancer analysis identified UBE2C as an oncogene in various tumor

**Fig 7:**
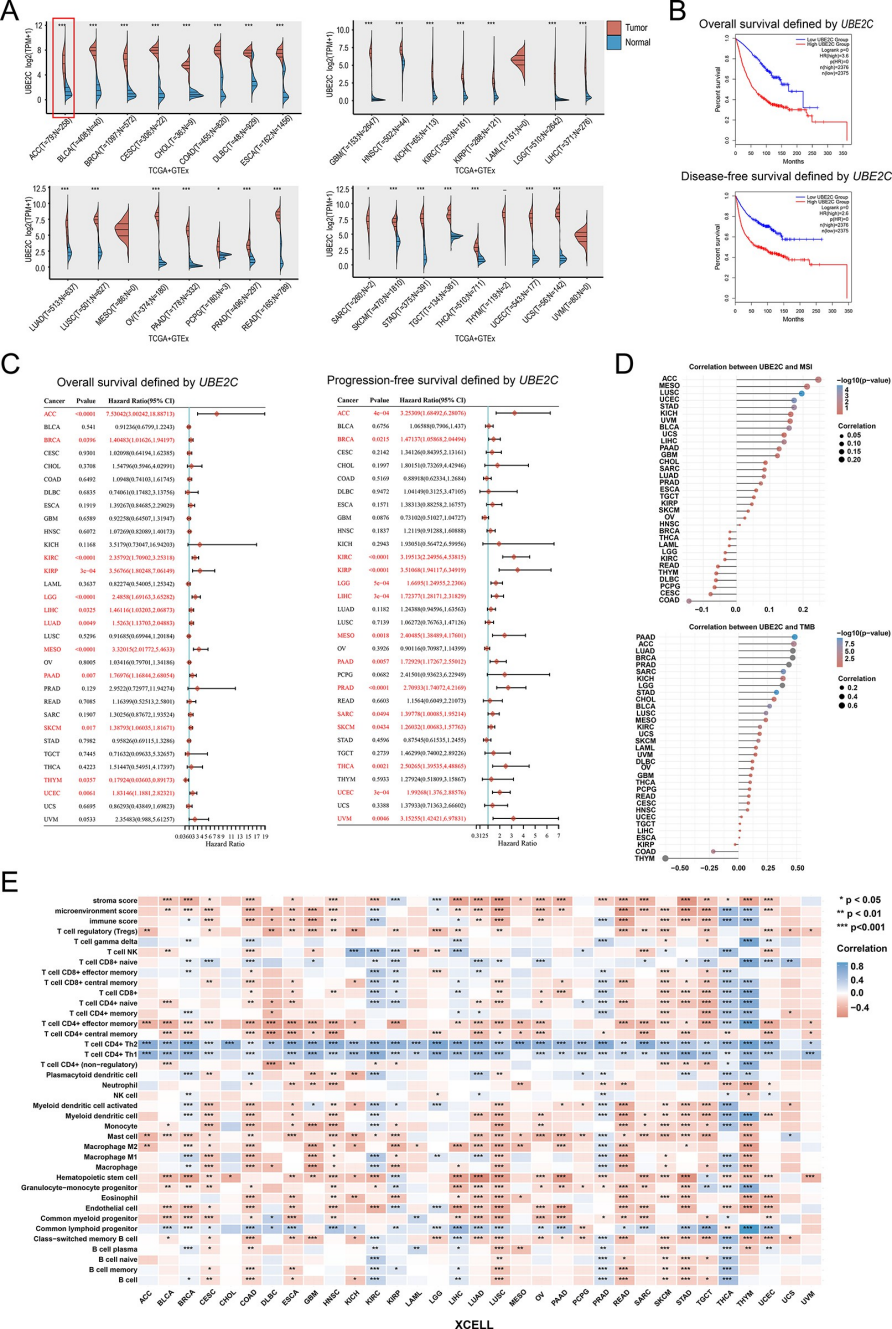
Pan-cancers analysis of UBE2C. (A) the different expression of UBE2C in 33 tumors. (B) the overall survival and disease-free survival defined by UBE2C in pan-cancer. (C) Forest plot: P-value, risk factor HR, and 95% confidence interval of the overall survival and disease-free survival defined by UBE2C in 33 tumors. (D) Spearman correlation analysis of UBE2C expression with MSI and TMB. The abscissa and the size of the dots in the figure represent the correlation coefficient, and the ordinate represent different tumors. The different colors represent the P-value; (E) Heatmap plot: spearman correlation analysis between immune infiltration scores and UBE2C expression in 33 tumor tissues using XCELL method. *P < 0.05, **P < 0.01, ***P < 0.001, the asterisk represents the degree of statistical significance (*P).

In this paper, pan-cancer analysis using TCGA dataset and GTEx dataset were all implemented by *ACLBI web-based Tools*. We found that UBE2C is almost overexpressed in all tumor including ACC, BLCA, BRCA, CESC, CHOL, COAD, DLBC, ESCA, GBM, HNSC, KICH, KIRC, KIRP, LGG, LIHC, LUAD, LUSC, OV, PAAD, PCPG, PRAD, READ, SARC, SKCM, STAD, TGCT, THCA, UCEC, and UCS by means of the comparison on UBE2C expression between the tumor and normal obtained from TCGA dataset and GTEx dataset, respectively (Fig 7A). Moreover, UBE2C expression will leads to poor prognosis in various tumors (Fig 7B and 7C). More importantly, we found that UBE2C expression is positively associated with hyperactive MSI as well as TMB, especially in ACC (Fig 7D). This may imply that UBE2C can be used as a predictor of the efficacy of immunotherapy for patients with ACC. The relationship between UBE2C expression and immune infiltration in various tumors were also explored. And we found that UBE2C overexpression was positive correlation with the infiltration of Th1, Th2 as well as negative correlation with the infiltration of Treg, M2 macrophages (Fig 7E). But the relation between the Th1/Th2 immune balance and ACC progression need more studies. In conclusion, we found that UBE2C was highly expressed in various tumors and strongly associated with poor prognosis, but also predicted the hyperactivity of MSI and TMB, especially in ACC.

### UBE2C knockdown weakened proliferation, migration, and invasion via inhibiting ACC cycle progression and EMT in vitro

**Fig 8:**
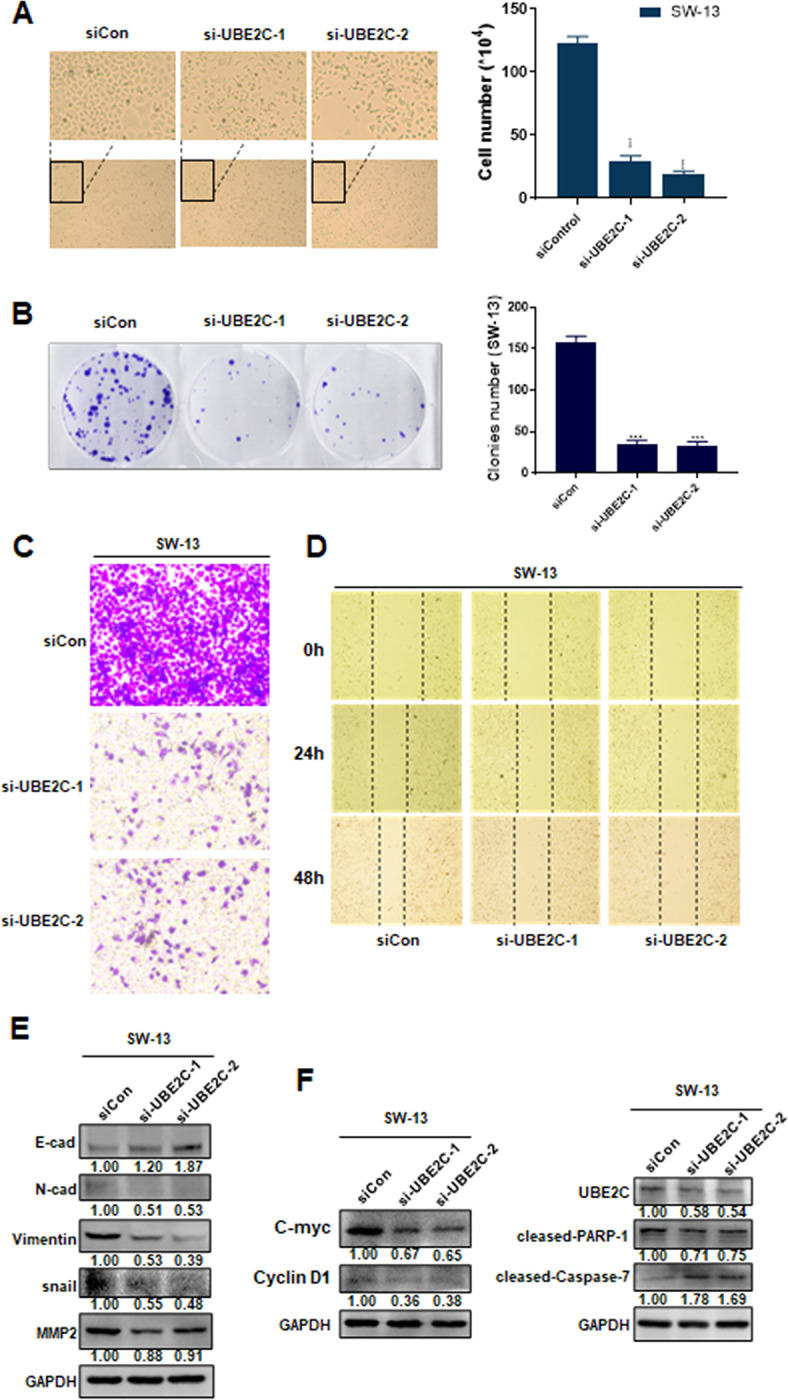
UBE2C knockdown weakened proliferation, migration, and invasion. (A) Morphological changes of SW-13 cells transfected with control or UBE2C siRNA. (B) SW-13 cells were transfected with control or UBE2C siRNA, and colonies were counted after 14 days. (C) SW-13 cells were transfected with control or UBE2C siRNA, and cell invasion was determined by transwell assay. (D) SW-13 cells were transfected with control or UBE2C siRNA, and cell migration was detected by wound healing assay. (E-F) Western blotting assay of the indicated proteins in SW-13 cells were transfected with UBE2C or control siRNA for 48 h. *P < 0.05, **P < 0.01, ***P < 0.001, the asterisk represents the degree of statistical significance (*P) (n = 3).

At first, we assessed the effects of UBE2C on adrenocortical carcinoma cell growth. Morphological changes of SW-13 cells showed that transfected with control or UBE2C siRNAs strongly inhibited proliferation of adrenocortical carcinoma cell (Fig 8A). The clone formation assays further verified the promoting effect of UBE2C on the proliferation of SW-13 cells (Fig 8B). To test our hypothesis that UBE2C is involved in tumor invasion, siRNAs were used to transiently silence UBE2C expression. And then the effect of UBE2C on migration and invasion in adrenocortical carcinoma cell were further investigated. Transwell assay results demonstrated that UBE2C siRNAs stimulation remarkably suppressed the invasion of adrenocortical carcinoma cell compared to treated with a scramble siRNA (Fig 8C). We assessed migration capacity through wound-healing assays in vitro with taking a picture and calculating the distance travelled at a 0-hour, 24-hour and 48-hour time point. Compared to SW-13 cells treated with a scramble siRNA, those receiving UBE2C siRNAs stimulation travelled a shorter distance (Fig 8D). For tumor invasion requiring both degradation of ECM components and cell migration, we performed western blotting and found that UBE2C knockdown indeed inhibited EMT of adrenocortical carcinoma cells, manifested as the decreased expression of N-cad, Vimentin, snail, MMP2 and increased expression of E-cadherin (Fig 8E). Moreover, western blotting indicated that UBE2C siRNAs can inhibit proliferation, inhibit damage repair of DNA, and induced apoptosis in ACC cells, characterized by the increased expression of cleased-Caspase-7 as well as the decreased expression of cleased-PARP-1, C-myc, cyclin D1 (Fig 8F). In conclusion, these results demonstrated that UBE2C knockdown can weaken proliferation, migration, and invasion of adrenocortical carcinoma cell via inhibiting cycle progression and EMT.

## Discussion

Adrenocortical carcinoma is a rare tumor with a very low incidence. However, patients with adrenocortical carcinoma usually have poor prognosis. When patients with adrenocortical carcinoma progressed from the stage I to the stage Ⅳ, the 5-year survival rate would be reduced by more than 60%. Unfortunately, only mitotane with limited therapeutical effects can be used for patients with advanced adrenocortical carcinoma or postoperative recurrence. Hence, it is a major challenge for the scientific community to develop more safe and more effective anti-ACC drugs. A new understanding of pathogenesis and an in-depth exploration of novel drug targets are urgently needed in ACC to find anti-cancer drugs.

It is helpful to excavate potential oncogene and to better integrate biological evidence for understanding the mechanism of tumor progression using web-based tools or database. We screened out 20 oncogenes including CDC20, NUF2, BUB1, CCNA2, LMNB1, PTTG1, FSCN1, MELK, KIF11, CEP55, CDCA3, TROAP, CDKN3, CCNB2, CDT1, AURKB, CDT1, AURKB, BIRC5, UBE2C, CENPM, KIF4A by analyzing TCGA data sets and GTEx data sets implemented by *ACLBI Web-based Tools*. We not only found these 20 oncogenes were highly expressed in tumor tissues, but also study the differential expression levels of these genes between tumor and normal. These results identified UBE2C as the significant DEG between tumor and normal.

Ubiquitin-proteasome system (UPS) is one of the major pathways for protein degradation through a series of steps such as substrate recognition, ubiquitin coupling and proteasome-ubiquitinated substrate degradation [19, 20]. Unlike autophagy, UPS primarily degrades single unfolded peptides with capacity of entering narrow channels of the proteasome [21]. Human UBE2C (also known as UBCH10) is located on chromosome 20q13.12 [22]. UBE2C can promote anaphase-promoting complex (APC/C) specifically combine with protein degradation pathway, and plays a key role in regulating the cell cycle, apoptosis, and transcriptional processes by catalyzing degradation of proteins [8, 23, 24]. APC/C, a multi-subunit complexes, can accelerate the mitotic progression from metaphase to anaphase by catalyzing the multi-ubiquitination of key regulatory factors in the cell cycle [25]. Hence, the abnormal expression of UBE2C will cause the hyperactivity of the APC/C-dependent ubiquitination in tumor cells, which accelerates the cell cycle and eventually leads to malignant transformation. It is well known that the KRAS^G12D^ target have ushered in a new era in the treatment of KRAS-mutant oncology drugs [26]. Remarkably, a study discovered that Kras^G12D^ mutation regulates the cell cycle by promoting the expression of UBE2C, which ultimately promotes tumor cell growth [11]. That validates UBE2C as a potential therapeutic target for lung cancer with KRAS^G12D^ mutations. Accumulating evidence also suggested that the high expression of UBE2C is strongly related to poor prognosis in patients with breast cancer [24, 27], lung adenocarcinoma [28] or gastric cancer [29]. On the one hand, based on the description of hallmarks of cancer proposed by Professor Douglas Hanahan [30], it has been confirmed that UBE2C can promote tumor growth [31], angiogenesis [32], tumor metastasis [33, 34], anchorage-independent growth [35], stemness [36], resist apoptosis [37, 38], induce immunosuppressive microenvironment [39], and enhance glycolytic activity [40, 41]. On the other hand, high expression of UBE2C will lead to treatment failure attributed to overactive anchorage-independent growth and reduced oxidative stress-induced cell apoptosis resulting in chemotherapy resistance [35], and reduce radiosensitivity [38], respectively. In short, a series of studies shown that UBE2C is highly expressed in tumors tissues and able to promote tumor progression.

In this study, we identified UBE2C as a potential oncogene after analyzing the TCGA dataset implemented by *ACLBI Web-based Tools*. Interestingly, we found that UBE2C only significantly expressed in patients with ACC stage Ⅲ and stage Ⅳ using *Xiantao Academic Web-based Tools*. Hence, patients with Advanced ACC or postoperative recurrence ACC are lack of effective treatment strategies. UBE2C is mainly expressed in advanced ACC, which may be a theoretical basis for solving these contradictions in ACC. Moreover, we evaluated the correlation between the expression levels of UBE2C and the function of m6A in ACC. At first, we identified the m6A methylation modification patterns in ACC mediated by 20 regulators using *ACLBI Web-based Tools* (Fig 4A). Accumulating evidence showed that more than 60% RNA modifications in mammalian cells are methylation modification, and m6A methylation is the most common, abundant, and conservative internal RNA modification [42]. M6A methylation can speed up transcription and participate in proteins translation and mRNA degradation [43–45]. m6A methylation modification can directly or indirectly regulate target genes, which can affect biological behaviors such as the proliferation, metastasis, and immune escape of tumor cells [46]. Hence, explaining the relationship between UBE2C and m6A regulators in ACC can enhance our understanding of the mechanism of UBE2C promoting the tumor development. In vitro experiments data showed that suppressing UBE2C expression can inhibit metastasis and proliferation, weaken DNA damage repair, and induce the apoptosis. Our preliminary results suggested that UBE2C can activate the C-myc signaling pathway to promote cell cycle progression as well as activate the snail signaling pathway to induce EMT in ACC. A study uncovered that PARP-1 can activate the snail signaling pathway to induce EMT in melanoma cells [47]. These results cued that the mechanism of UBE2C promoting proliferation and metastasis of ACC will be more complicated, and further investigations are still needed to clarify this important issue.

## Conclusions

In summary, the present study demonstrates that UBE2C is strongly correlation with poor prognosis in patients with ACC via promoting cell cycle progression and EMT. This study not only uncovers the mechanism of UBE2C in promoting ACC proliferation and metastasis but also provides a novel rationale for developing UBE2C as a potential molecular target for the treatment of advanced ACC.

## Supporting information

S1 Table The DEGs of the patients with ACC. The differentially expressed genes between patients with ACC and normal were analyzed. RNA-sequencing expression profiles of 79 patients with ACC were downloaded from the TCGA database. And RNA-sequencing expression profiles of 258 normal were downloaded from GTEx database. These data were analyzed using R packages implemented by R version 4.0.3 in ACLBI Web-based Tools (https://www.aclbi.com/). “LogFC ≥ 2 or LogFC ≤ -2, P-value < 0.05” was defined as a screening criterion for identifying differentially expressed genes.
(XLSX)

S2 Table 20 candidate genes in ACC were identified. The intersection of four gene clusters was analyzed including DFS-relates genes, OS-relates genes, differentially expressed genes (DEGs), and highly expressed genes.
(XLSX)

S3 Table UBE2C was the most significant DEG between ACC and normal. RNA-sequencing expression profiles of 79 patients with ACC were downloaded from the TCGA database. And RNA-sequencing expression profiles of 258 normal were downloaded from GTEx database. These data were analyzed using R packages implemented by R version 4.0.3 in ACLBI Web-based Tools (https://www.aclbi.com/).
(XLSX)

S4 Table UBE2C expression within the ACC dataset. UBE2C expression and corresponding clinical information of 79 patients with ACC were described in detail.
(XLSX)

S5 Table G1 group contained 16 significantly overexpressed m6A regulators compared with G2 group. The result showed that G1 group (above 75th percentile of UBE2C expression) contained 16 significantly overexpressed m6A regulators compared with G2 group (below 25th percentile of UBE2C expression).
(XLSX)

S6 Table DEGs between G1 group and G2 group. The result showed the differentially expressed genes between G1 group (above 75th percentile of UBE2C expression) and G2 group (below 25th percentile of UBE2C expression).
(XLSX)

S7 Table A cluster containing 4 genes related to cell cycle by intersecting the two data sets. The candidate proteins obtained from STRING database; and the significant DEGs between G1 group (above 75th percentile of UBE2C expression) and G2 group (below 25th percentile of UBE2C expression) were analyzed from the TCGA dataset. “LogFC ≥ 2 or LogFC ≤ -2, P-value < 0.05” was defined as a screening criterion.
(XLSX)

S1 Raw images (PDF)

## Abbreviations

UBE2C: Ubiquitin Conjugating Enzyme 2C
DEGs: differentially expressed genes
EMT: epithelial–mesenchymal transition
mRNAsi: mRNA expression-based stemness index
ACC: Adrenocortical carcinoma
BLCA: Bladder Urothelial Carcinoma
BRCA: Breast invasive carcinoma
CESC: Cervical squamous cell carcinoma and endocervical adenocarcinoma
CHOL: Cholangio carcinoma
COAD: Colon adenocarcinoma
DLBC: Lymphoid Neoplasm Diffuse Large B-cell Lymphoma
ESCA: Esophageal carcinoma
GBM: Glioblastoma multiforme
HNSC: Head and Neck squamous cell carcinoma
KICH: Kidney Chromophobe
KIRC: Kidney renal clear cell carcinoma
KIRP: Kidney renal papillary cell carcinoma
LGG: Brain Lower Grade Glioma
LIHC: Liver hepatocellular carcinoma
LUAD: Lung adenocarcinoma
LUSC: Lung squamous cell carcinoma
OV: Ovarian serous cystadenocarcinoma
PAAD: Pancreatic adenocarcinoma
PCPG: Pheochromocytoma and Paraganglioma
PRAD: Prostate adenocarcinoma
READ: Rectum adenocarcinoma
SARC: Sarcoma
SKCM: Skin Cutaneous Melanoma
STAD: Stomach adenocarcinoma
TGCT: Testicular Germ Cell Tumors
THCA: Thyroid carcinoma
UCEC: Uterine Corpus Endometrial Carcinoma
UCS: Uterine Carcinosarcoma
