# Supplementary material for: System analysis identifies UBE2C as a novel oncogene target for adrenocortical carcinoma
Source: PLoS One. 2023 Aug 3;18(8):e0289418. doi: 10.1371/journal.pone.0289418 (PMC10399895; doi:10.1371/journal.pone.0289418)

Figure 8E：raw_images

Vimentin

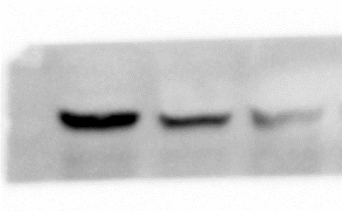

Snail

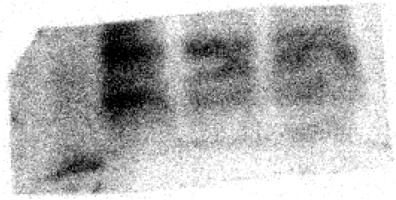

N-cad

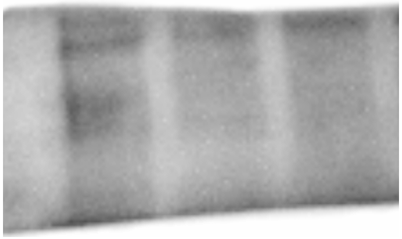

E-cad

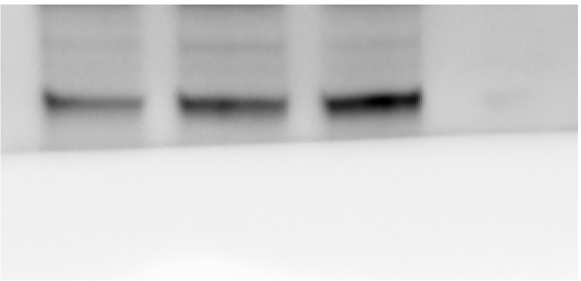

MMP2

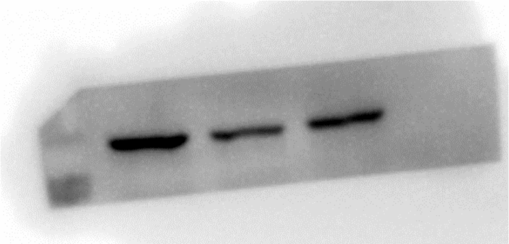

GAPDH

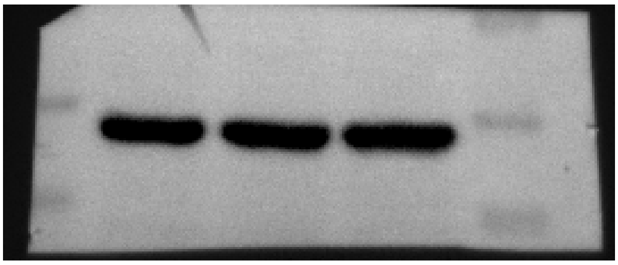

Figure 8F：raw_images

UBE2C

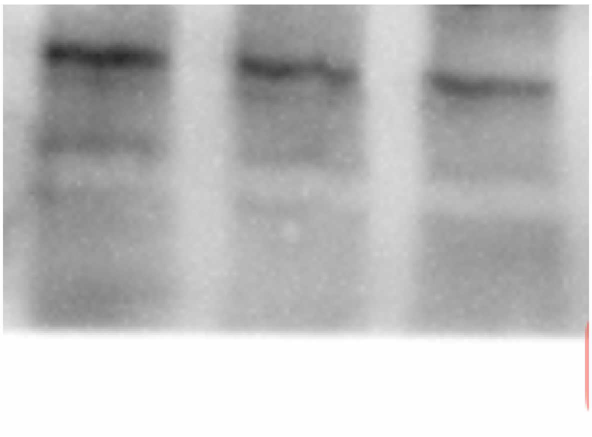

GAPDH

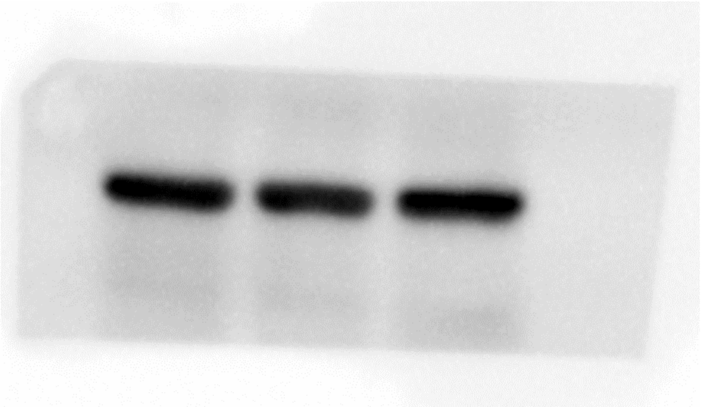

GAPDH

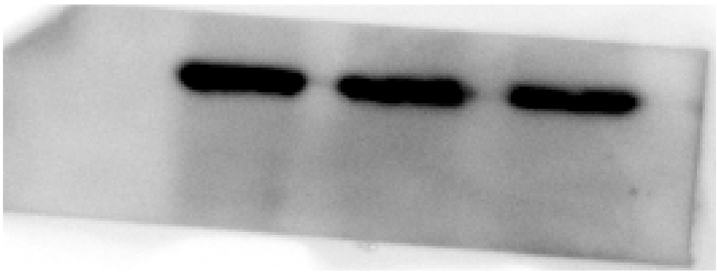

Cyclin D1

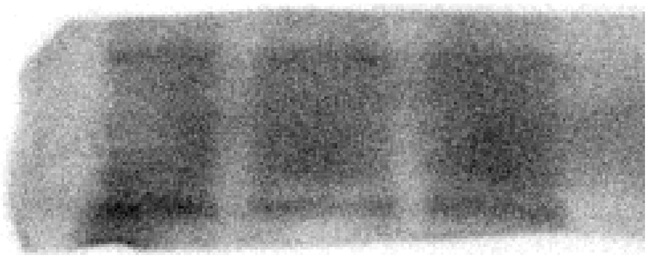

cleased-PARP-1

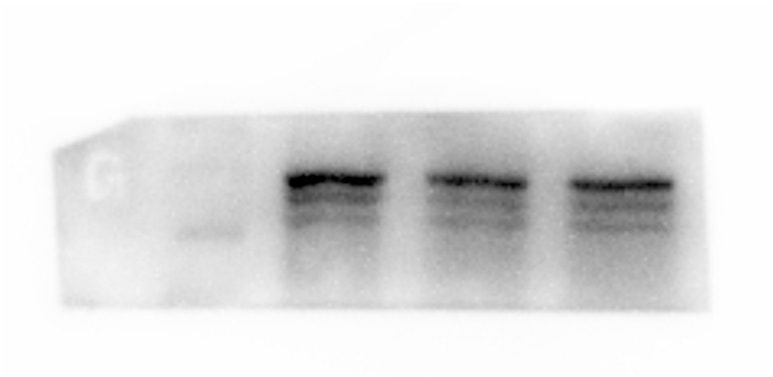

C-myc

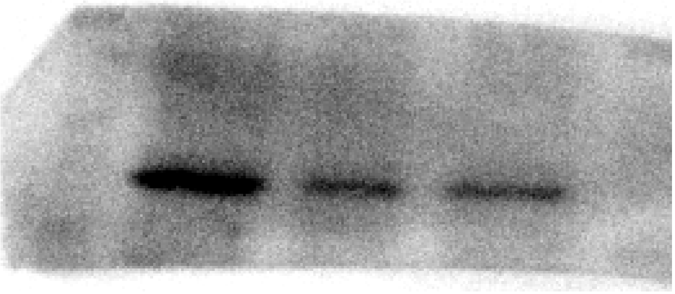

cleased-Caspase-7

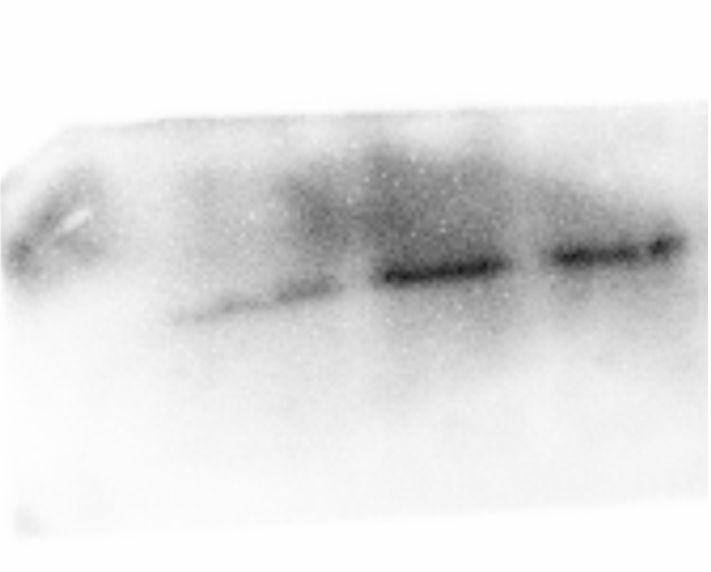

Supplement: S1 Raw images — (PDF) [file pone.0289418.s008.pdf]
